# Inhibition of the DNA Damage Pathway by a Telomere-Binding Protein

**DOI:** 10.1371/journal.pbio.0020270

**Published:** 2004-08-17

To maintain the integrity of their genetic content, cells closely monitor the state of their chromosomal DNA. Any break or pairing anomaly in the double helix is perceived as damage that must be repaired. Mechanisms that sense DNA damage enlist the help of cell cycle checkpoint proteins, which stall cell division until the damage has been repaired. The exposed ends of linear chromosomes of eukaryotic cells (cells with nuclei) resemble the double-strand breaks of damaged DNA, which should make them vulnerable to unnecessary manipulations by repair enzymes. But chromosomes have special DNA sequences at their tips, called telomeres, that are coated with telomere-binding proteins that appear to protect ends from the unwanted attentions of repair enzymes. For instance, removing a telomere-specific DNA-binding protein, called TRF2, from telomeres leads to a rapid attack on chromosome tips, associated with the activation of the DNA damage pathway. The biochemical basis for TRF2's shielding effects remains obscure.

In this issue of *PLoS Biology*, Jan Karlseder et al. propose that TRF2 is a direct inhibitor of an early mediator of the DNA damage signal. Their observations offer new insights into how telomeres resist the inappropriate interventions of the DNA repair machinery.

Ataxia telangiectasia mutated (ATM) kinase is an enzyme that induces the activation of DNA repair enzymes as well as regulators of the cell cycle and apoptosis. Its enzymatic function is triggered by DNA damage sensors. Several observations suggest an antagonism between TRF2 and ATM: ATM is activated in aging cells with shortened telomeres and participates in ushering the cell into replicative senescence. (Cells divide only so many times during their lives before forgoing the process altogether.) In contrast, TRF2 overexpression protects shortened telomeres from decay and delays cell entry into senescence.

Here the authors examine the effect of TRF2 overexpression in cells subjected to radiation-induced DNA damage. This treatment is expected to lead to cell cycle arrest, an outcome mediated for the most part by ATM activation. Irradiated cells that overexpress TRF2, however, continue to enter cell division. Known targets of ATM's enzymatic activity are activated to a lesser degree in these cells, which harbor only about half the amount of active ATM detected in controls. This suggests that TRF2 may interfere with the DNA damage pathway early on, when ATM is activated.

Do these findings reflect a direct interaction between ATM and TRF2? Karlseder et al. suggest they might. Though only a small fraction of TRF2 associates with ATM in normal cells, ATM and TRF2 proteins can form a complex in a test tube. The region of the ATM protein that interacts with TRF2 is the very region required for ATM's activation by DNA damage sensors. The authors propose that TRF2 binds to inactive ATM, and in so doing, prevents ATM's transition to an active state.

The authors integrate their results in an elegant proposal that relates a cell's health to the length of its telomeres. They have previously shown that TRF2 binds another protein called Mre11, a DNA damage sensor known to activate ATM. Thus, by inhibiting ATM, TRF2 may nip in the bud any misguided attempts by Mre11 to “repair” DNA breaks in telomeres. But in aging cells, whose shortened telomeres no longer retain large amounts of TRF2, ATM activation, no longer muffled, eventually allows the onset of senescence. While TRF2 is abundant in normal cells, the authors note that its strict association with telomeres should locally limit its effect on ATM, leaving the DNA damage pathway intact at other chromosomal locations.

